# Heterotropic Activation of Cytochrome P450 3A4 by Perillyl Alcohol

**DOI:** 10.3390/pharmaceutics16121581

**Published:** 2024-12-11

**Authors:** Ji Hyeon Ryu, Jieun Yu, Jang Su Jeon, Seongyea Jo, Soo Min Lee, Hyemin Kim, Han-Jin Park, Soo Jin Oh, Sang Kyum Kim

**Affiliations:** 1 College of Pharmacy, Chungnam National University, Daejeon 34134, Republic of Korea; jihyeon.ryu@kitox.re.kr (J.H.R.); rhdwn0117@hanmail.net (J.Y.); navijang2@gmail.com (J.S.J.); 2 Center for Biomimetic Research, Division of Advanced Predictive Research, Korea Institute of Toxicology, Daejeon 34114, Republic of Korea; seongyea.jo@kitox.re.kr (S.J.); hyeminkim@kitox.re.kr (H.K.); hjpark@kitox.re.kr (H.-J.P.); 3 Department of Medical Science, Asan Medical Institute of Convergence Science and Technology, Asan Medical Center, University of Ulsan College of Medicine, Seoul 05505, Republic of Korea; lsumin052@gmail.com

**Keywords:** heterotropic activation, CYP3A4, perillyl alcohol, human hepatic organoids, drug interaction

## Abstract

**Background/Objectives**: Perillyl alcohol (POH), a monoterpene natural product derived from the essential oils of plants such as perilla (*Perilla frutescens*), is currently in phase I and II clinical trials as a chemotherapeutic agent. In this study, we investigated the effect of POH on cytochrome P450 (CYP) activity for evaluating POH–drug interaction potential. **Methods**: The investigation was conducted using pooled human liver microsomes (HLMs), recombinant CYP3A4 (rCYP3A4) enzymes, and human pluripotent stem cell-derived hepatic organoids (hHOs) employing liquid chromatography-tandem mass spectrometry. **Results**: POH inhibited the activities of CYP2A6 and CYP2B6 with K_i_ of 6.35 and 3.78 μM, respectively, whereas it stimulated CYP3A4 activity in pooled HLMs incubated with midazolam (MDZ). In a direct CYP inhibition assay using HLMs, activities of CYP2C9, CYP2C19, and CYP2E1 were also inhibited by POH, with IC_50_ values greater than 50 μM, but those of CYP1A2, CYP2C8, CYP2D6, and CYP3A4 (testosterone) were not significantly inhibited. In pooled HLMs, the V_max_/K_m_ value of 1′-hydroxy MDZ, but not that of 4-hydroxy MDZ, was increased 2.7-fold by 100 μM POH compared with that in the absence of POH. Moreover, stimulation of MDZ 1′-hydroxylation by CYP3A4 was observed in hHOs and rCYP3A4 with cytochrome *b*_5_ but not rCYP3A4 without cytochrome *b*_5_. Furthermore, activation of CYP3A4-mediated metabolism by POH was observed in HLMs incubated with fimasartan but not atorvastatin, buspirone, donepezil, nifedipine, or tadalafil, suggesting a substrate-dependent activation of CYP3A4 by POH. **Conclusions**: POH inhibits CYP2A6 and CYP2B6, but it activates CYP3A4. These findings underscore the need for further evaluation of the interactions of clinical drugs with POH.

## 1. Introduction

Cytochrome P450 (CYP) enzymes belong to a superfamily of hemoproteins that are involved in metabolizing endogenous compounds as well as xenobiotics [1]. CYP isoforms are the most important drug-metabolizing enzymes, which metabolize approximately 80% of all clinically used therapeutic agents [2]. CYP enzymes, including CYP3A4, can be induced or inhibited by various xenobiotics, such as drugs, environmental chemicals, and phytochemicals [3]. This induction or inhibition leads to clinically significant drug–drug interactions, affecting the efficacy and safety of drugs. Moreover, CYP enzymes can exhibit cooperativity in their metabolic activity, which can be homotropic (modulated by the substrate itself) or heterotropic (modulated by different effectors or compounds), adding a layer of complexity to how these enzymes process substrates [4].

Perillyl alcohol (POH) is a monoterpene found in perilla (*Perilla frutescens*), the peel of citrus fruits, and other plants [5]. POH is known to be a major metabolite of limonene (LMN) [6]. LMN exists in two optical isomers, (+) and (−). The optical isomeric forms are maintained in the metabolic process [7]. In humans, LMN is metabolized to POH by CYP2C9 and CYP2C19, and POH is sequentially metabolized to perillaldehyde (PAH) and then to perillic acid (PA) [6,8]. LMN, POH, and PAH are fragrant ingredients commonly used in manufacturing, household uses, and cosmetics [9,10]. They have also been approved by the U.S. Food and Drug Administration as food additives and are mainly used as flavoring agents [10,11,12]. POH is listed as generally recognized as safe by the U.S. Food and Drug Administration and approximately maximal use level of POH was reported as 10 ppm [13]. Additionally, these monoterpenes exhibit various biological activities, particularly anti-cancer properties. Among the derivatives of LMN, POH exhibits the most potent antiproliferative effects [7]. For this reason, several phase I and II clinical trials have been conducted using POH as a potential anti-cancer agent [14]. However, there are no reports of the drug interaction potential of POH and metabolites.

In this study, we investigate the effect of POH, along with that of its precursor and metabolites, on CYP activities to evaluate their drug–drug interaction potential. In direct CYP inhibition assay using pooled human liver microsomes (HLMs), CYP2A6, CYP2B6, and CYP2C19 were inhibited by (−)-PAH and, to a lesser extent, by (+)-PAH. CYP2A6 and CYP2B6 were also inhibited by (−)-POH. Interestingly, an enhancement of CYP3A4-mediated midazolam (MDZ) 1′-hydroxylation by POH was observed in pooled HLMs. To clarify the activation of CYP3A4, kinetic studies on MDZ hydroxylation were conducted with pooled HLMs. We also confirmed similar increases in MDZ 1′-hydroxylation activities by POH using human pluripotent stem cell-derived hepatic organoids (hHOs) [15,16] and recombinant CYP3A4 (rCYP3A4) with cytochrome *b*_5_ which can modulate CYP activity as a redox partner of CYP [17,18,19,20]. Furthermore, the effect of POH on the metabolism of other CYP3A4 substrates was examined in pooled HLMs.

## 2. Materials and Methods

### 2.1. Chemical and Reagents

(+)-LMN, (−)-LMN, (−)-PAH, and (−)-PA were bought from Sigma-Aldrich (St. Louis, MO, USA). (−)-POH and (+)-PAH were purchased from Toronto Research Chemicals Inc. (Toronto, ON, Canada). Figure S1 illustrates their chemical structures, highlighting the chiral center. All chemicals and reagents utilized in the experiments were either of analytical grade or the highest commercial quality.

### 2.2. Direct CYP Inhibition Assay

Stock solutions of each test compound (as an inhibitor) were prepared at 10 mM in dimethylsulfoxide (DMSO; Sigma-Aldrich) and diluted to the working concentration using DMSO. The working solution was then spiked into 0.1 M potassium phosphate buffer (PPB) at pH 7.4. The final DMSO concentration in the reaction mixture was 0.5%. LMN, POH, PAH, or PA was pre-incubated at 37 °C for 5 min with 0.2 mg/mL HLMs (Corning Gentest, Corning, NY, USA) and two different CYP isoform-specific probe substrate cocktail sets (set A: 50 μM phenacetin (Sigma-Aldrich) for CYP1A2, 5 μM coumarin (Sigma-Aldrich) for CYP2A6, 2 μM amodiaquine (Sigma-Aldrich) for CYP2C8, 100 μM S-mephenytoin (Cayman, Ann Arbor, MI, USA) for CYP2C19, 5 μM dextromethorphan (Sigma-Aldich) for CYP2D6, and 5 μM MDZ (Bukwang Pharma Co., Seoul, Republic of Korea) for CYP3A4; set B: 50 μM bupropion (Sigma-Aldrich) for CYP2B6, 100 μM tolbutamide (Sigma-Aldrich) for CYP2C9, 50 μM chlorzoxazone (Sigma-Aldrich) for CYP2E1, and 50 μM testosterone (Sigma-Aldrich) for CYP3A4) in 0.1 M PPB at pH 7.4). Based on the linearity of the reaction rate for incubation time, non-specific binding, and metabolite quantification limits, 0.2 mg/mL of HLMs was incubated with substrates for 10 min [21]. In all experiments, the substrates were dissolved and serially diluted with acetonitrile to the required concentrations. The final acetonitrile concentrations for the cocktail incubation conditions were 1.0% (*v*/*v*) (set A) and 0.5% (*v*/*v*) (set B). The enzyme reactions were initiated by adding 1 mM reduced form of β-nicotinamide adenine dinucleotide phosphate (NADPH; Toronto Research Chemicals Inc., Toronto, ON, Canada) and incubated for 10 min. The incubation mixtures were quenched by adding 200 μL of ice-cold acetonitrile containing 100 nM carbamazepine (Sigma-Aldrich) or 300 nM 4-methyl-umbelliferone (Sigma-Aldrich) as an internal standard for analytes detected in positive mode or for 6-hydroxychlorzoxazone detected in negative mode, respectively. Samples were vigorously vortexed and centrifuged at 4 °C for 20 min at 3000× *g*. The supernatants were analyzed using liquid chromatography-tandem mass spectrometry (LC-MS/MS). The experiment was performed using three separate samples.

### 2.3. Metabolism-Dependent Inhibition Assay

The experiments for determining metabolism-dependent inhibition were conducted utilizing a non-dilution method [21]. Pre-incubation mixtures (180 μL) consisted of 0.1 M PPB (pH 7.4), 0.2 mg/mL HLMs, 1 mM NADPH, and various concentrations of (–)-POH or (–)-PAH (0–50 µM for (–)-POH; 0–20 µM for (–)-PAH, respectively). The pre-incubation was initiated by the addition of 1 mM NADPH and incubation for 30 min. Subsequently, final incubation was performed with the addition of a substrate (5 μM coumarin or 50 μM bupropion) in the presence of NADPH for 10 min; the total volume of the mixture was 200 μL. The reactions were terminated by adding 200 μL of ice-cold acetonitrile containing 100 nM carbamazepine as an internal standard. Mixtures were subjected to centrifugation at 3000× *g* for 20 min at 4 °C, and the supernatants were analyzed using LC-MS/MS. The experiment was performed using three separate samples.

### 2.4. Determination of CYP Inhibition Mode

The incubation mixtures (200 μL) comprised 0.1 M PPB (pH 7.4), 0.2 mg/mL HLMs, 1 mM NADPH, (–)-POH or (–)-PAH at various concentrations (0–50 µM for (–)-POH; 0–20 µM for (–)-PAH, respectively) in the presence of coumarin (1.25–10 μM) or bupropion (12.5–100 μM). A 5 min pre-incubation was performed prior to initiating reactions with the addition of 1 mM NADPH. After incubation at 37 °C for 10 min, 200 μL of ice-cold acetonitrile containing 100 nM carbamazepine as an internal standard was added to terminate the reaction. The samples were agitated and centrifuged at 4 °C for 20 min at 3000× *g*. The supernatants were transferred to a 96-well plate for LC-MS/MS analysis. The experiment was performed using three separate samples.

### 2.5. Determination of Kinetic Parameters

The pre-incubation was carried out in 0.1 M PPB (pH 7.4), 0.2 mg/mL pooled HLMs, and (–)-POH (0–100 μM) at concentrations ranging from 0.5 to 20 μM of MDZ for 5 min. The reactions were initiated with 1 mM NADPH and then terminated with 200 μL of ice-cold acetonitrile containing 100 nM carbamazepine as an internal standard after 10 min incubation at 37 °C. The supernatants obtained after centrifuging at 3000× *g* for 20 min at 4 °C were injected into LC-MS/MS for analysis. The experiment was performed using four separate samples.

### 2.6. Enzyme Activity Assay Using rCYP3A4

Incubation mixtures (final volume, 200 μL) contained 0.1 M PPB (pH 7.4), 50 pmol/mL rCYP3A4 with or without cytochrome *b*_5_, 1 mM NADPH, 5 μM MDZ, and POH (0–100 μM). After preincubating for 5 min, the reactions were initiated by adding 1 mM NADPH and incubated for 10 min. Reactions were terminated by introducing 200 μL of ice-cold acetonitrile containing 100 nM carbamazepine as the internal standard. After centrifugation at 4 °C for 20 min at 3000× *g*, the supernatants were subjected to LC-MS/MS analysis. The experiment was performed using three separate samples.

### 2.7. POH Concentration-Dependent MDZ Hydroxylation in hHOs

To assess the effect of POH on the kinetics of the formation of 1′-hydroxymidazolam (1′-OH-MDZ) and 4-hydroxymidazolam (4-OH-MDZ), 5 μM MDZ was incubated with POH (0–100 μM) in hHOs for 24 h at 37 °C in a CO_2_ incubator. Samples were incubated in 24-well culture plates, with each well containing a total volume of 0.7 mL. At 2, 4, 6, and 24 h, 70 μL of medium was sampled, and reaction termination was achieved by adding an equal volume of ice-cold acetonitrile containing 100 nM carbamazepine as an internal standard. The supernatants were analyzed using LC-MS/MS after centrifugation at 10,000× *g* for 10 min at 4 °C. The hHOs used in the experiments were cultured following the methods described in the publications of Kim et al. [15] and Kim et al. [16]. Data were normalized for protein concentrations measured using the Pierce™ BCA Protein Assay Kit (Thermo Fisher Scientific, Waltham, MA, USA) following the manufacturer’s instructions. The experiment was performed using four separate samples.

### 2.8. Effect of POH on the Metabolism of Other CYP3A4 Substrates

Incubation mixtures (final volume, 200 μL) contained 0.1 M PPB (pH 7.4), 0.2 mg/mL HLMs, 1 mM NADPH, POH at various concentrations, and other CYP3A4 substrates (30 μM atorvastatin, 8 μM buspirone, 35 μM donepezil, 30 μM fimasartan (FMS), 25 μM nifedipine, or 5 μM tadalafil). The substrates were applied at a concentration that approximated their K_m_ values [22,23,24,25,26,27,28]. The reactions were preincubated for 5 min before being initiated with the addition of 1 mM NADPH. After 10 min incubation, reactions containing atorvastatin, buspirone, FMS, or nifedipine were terminated by the addition of 200 μL of ice-cold acetonitrile with 100 nM carbamazepine as an internal standard. For terminating reactions with donepezil or tadalafil, 200 μL of ice-cold methanol containing 0.5% (*v*/*v*) formic acid and 1 μM carbamazepine as internal standard was added to samples. Centrifugation of the samples was performed at 3000× *g* for 20 min at 4 °C, and the resulting supernatants were analyzed through LC-MS/MS. The experiment was performed using three separate samples.

### 2.9. Analytical Methods

For analyzing metabolites of CYP isoform selective substrates, FMS, and its metabolites, the samples were analyzed using a Prominence UFLC system with a parallel LC-20AD-XR pump, autosampler, and column oven (Shimadzu, Kyoto, Japan). Separation was achieved using an Atlantis dC18 column (2.1 mm × 50 mm, 3 μm; Waters, Milford, MA, USA) in combination with a SecurityGuard™ C18 guard column (2.0 mm × 4.0 mm; Phenomenex, Torrance, CA, USA) and the sample injection volume was 10 μL. The temperature of the column was controlled at 30 °C, and the auto-sampler compartment was set to 10 °C throughout the analysis. Deionized water (A) and acetonitrile (B), each supplemented with 0.1% (*v*/*v*) formic acid, were used as the mobile phases. The HPLC was run at a flow rate of 0.4 mL/min with the following linear gradient profile: 0 min, 0% B; 1 min, 0% B; 1.1 min, 45% B; 4 min, 50% B; 4.1 min, 95% B; 7 min, 95% B; 7.01 min, 0% B; 8 min, 0% B, followed by 2 min re-equilibration step. The accuracy, precision, and linearity of analytical methods used in this study were reported in our previous study [21]. Table S1 shows lower limits of quantification of CYP metabolites.

For analysis of donepezil, tadalafil, and their metabolites, the samples were analyzed using the LC system described above. Separation was achieved at 40 °C using ZORBAX Eclipse XDB-C8 (4.6 mm × 150 mm, 5 μm; Agilent Technologies, Santa Clara, CA, USA) in combination with a SecuityGuard™ C18 guard column (2.0 mm × 4.0 mm; Phenomenex), with a sample injection volume of 10 μL. Deionized water (A) and acetonitrile (B), each supplemented with 0.1% (*v*/*v*) formic acid, were used as the mobile phases. The HPLC was run at a flow rate of 0.5 mL/min with the following linear gradient profile: 0 min, 10% B; 0.1 min, 10% B; 7 min, 35% B; 10 min, 100% B; 15 min, 100% B, followed by 2 min re-equilibration step. Figure S2 shows representative LC-MS/MS chromatograms of CYP-mediated metabolite standards and internal standards.

The detection was conducted using an API 4000 mass spectrometer featuring a Turbo V™ IonSpray source (SCIEX, Framingham, MA, USA). The operational parameters included an ionization voltage of 5500 V in positive-ion mode and −4500 V in negative-ion mode, an ion source temperature of 600 °C, and gas pressures set as follows: nebulizer and heater gases at 50 psi each, and curtain gas at 20 psi. The samples were analyzed in multiple reaction monitoring (MRM) mode. More detailed MRM parameters of analytes are listed in Supplementary Materials (Table S2).

### 2.10. Data Analysis

The LC-MS/MS data were acquired and processed using Analyst™ software (version 1.6.2; Applied Biosystems, Foster City, CA, USA). Metabolite peak areas were normalized by calculating their ratio to the peak area of the internal standard for each test substance concentration.

The CYP-mediated activities in the presence of test substances were expressed as percentages of the corresponding control values. The IC_50_, representing enzyme inhibition, was determined by fitting a sigmoid curve and applying the Hill equation through nonlinear regression (least-squares best-fit modeling) to the percentage control activity versus test substance concentration, analyzed using GraphPad Prism version 8.0.1 (GraphPad Software Inc., San Diego, CA, USA). IC_50_ fold-shift values were calculated by dividing the IC_50_ value obtained after pre-incubation without NADPH by the IC_50_ value obtained after 30 min pre-incubation with NADPH.

Data acquired from the evaluation of enzyme inhibition using various concentrations of substrates and test compounds were fit to different models (competitive, noncompetitive, uncompetitive, or mixed), and inhibition constant (K_i_) and goodness of fit values were obtained using GraphPad Prism. The appropriate type of inhibition was selected based on statistical goodness of fit tests including Akaike’s information criterion (AICc) with correction for small sample size and the standard deviation of the residuals (Sy.x).

Kinetic parameters were estimated using GraphPad Prism to plot activities against the log of MDZ concentration in the presence of (–)-POH.

## 3. Results

### 3.1. Direct CYP Inhibition by LMN, POH, PAH, and PA in HLMs

**Figure 1:**
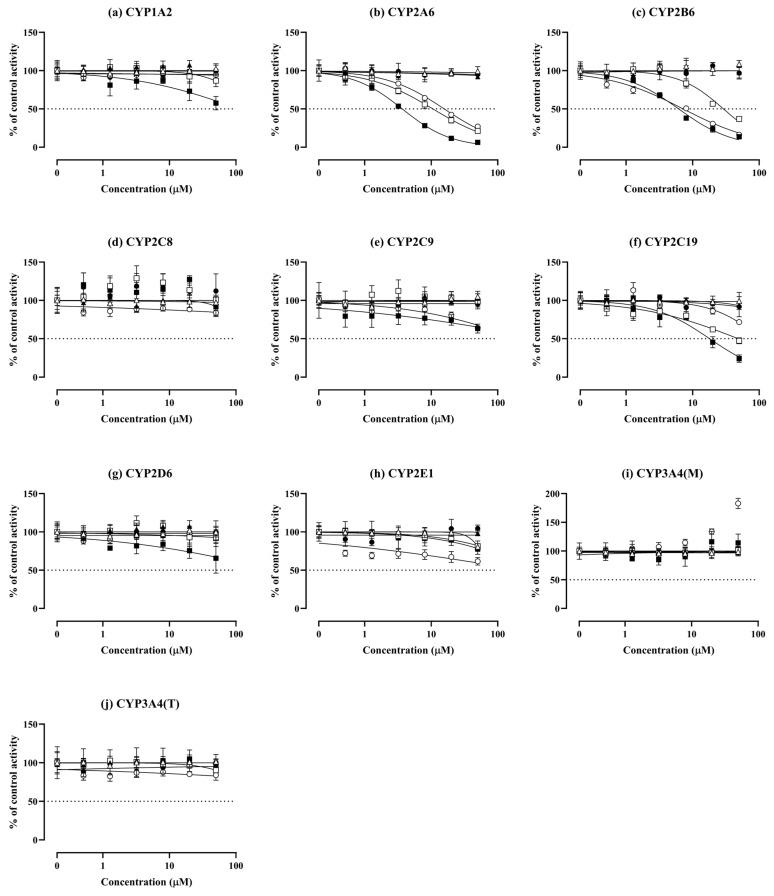
Effect of (+)-limonene (LMN, △), (−)-LMN (▲), (+)-perillaldehyde (PAH, □), (−)-PAH (■), (−)-perillyl alcohol (POH, ○), and (−)-perillic acid (PA, ●) on cytochrome P450 (CYP) 1A2 (**a**), CYP2A6 (**b**), CYP2B6 (**c**), CYP2C8 (**d**), CYP2C9 (**e**), CYP2C19 (**f**), CYP2D6 (**g**), CYP2E1 (**h**), CYP3A4 (midazolam (MDZ)) (**i**), and CYP3A4 (testosterone) (**j**) in pooled human liver microsomes (HLMs). The activity is expressed as a percentage of remaining activity compared with the control containing no inhibitor. Data are presented as mean ± SD of values for three separate samples.

The effects of LMN, POH, PAH, and PA on nine CYP isoforms (CYP1A2, CYP2A6, CYP2B6, CYP2C8, CYP2C9, CYP2C19, CYP2D6, CYP2E1, and CYP3A4) were evaluated in pooled HLMs (Table 1 and Figure 1). Table S3 shows the IC_50_ values of known CYP isoform-selective inhibitors, which fall within the reference ranges [21]. Activities of CYP isoforms were not significantly inhibited by LMN and PA. CYP2A6, CYP2B6, and CYP2C19 were inhibited by (−)-PAH and, to a lesser extent, by (+)-PAH (Figure 1b,c,f). (−)-POH also exhibited inhibitory activity against CYP2A6 and CYP2B6 (Figure 1b,c). The activities of CYP2C9, CYP2C19, and CYP2E1 were weakly inhibited by (−)-POH with IC_50_ values greater than 50 μM (Figure 1e,f,h), while CYP1A2, CYP2C8, CYP2D6, and CYP3A4 (TST) were not significantly inhibited by (−)-POH (Figure 1a,d,g,j). Interestingly, at 50 μM (−)-POH, the formation of 1′-OH-MDZ was increased 1.83-fold relative to control without (−)-POH (Figure 1i).

### 3.2. Metabolism-Dependent Inhibition Assay

**Figure 2:**
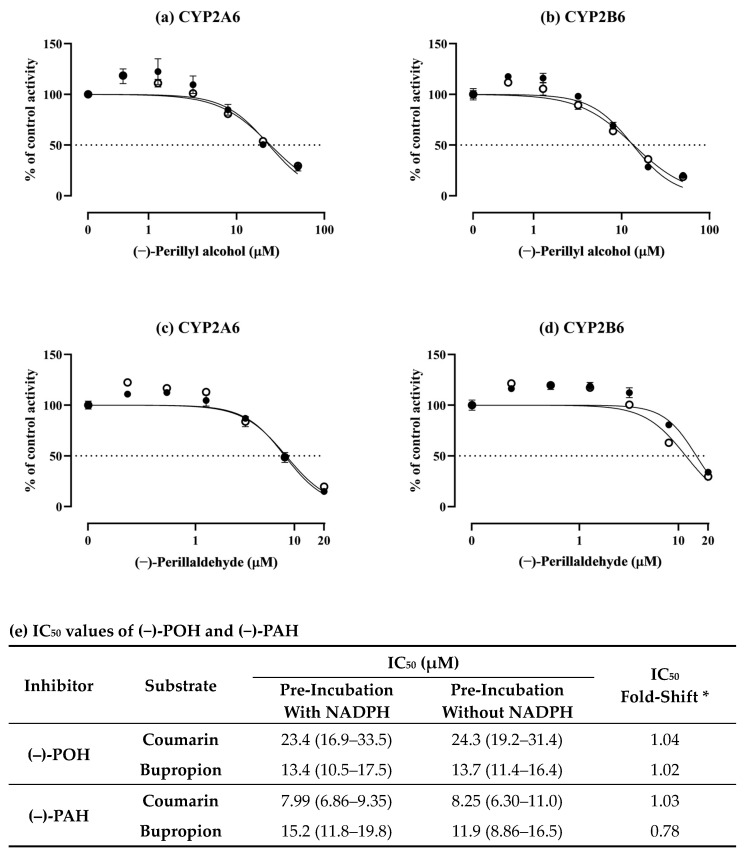
Inhibitory effects of (−)-POH and (−)-PAH on CYP2A6 and CYP2B6 after pre-incubation for 30 min with (●) or without (○) NADPH in pooled HLMs. (**a**,**b**) Inhibition curves for the inhibition of CYP2A6 and CYP2B6 by (−)-POH (0, 0.512, 1.28, 3.2, 8, 20, or 50 μM). (**c**,**d**) Inhibition curves for the inhibition of CYP2A6 and CYP2B6 by (−)-PAH (0, 0.205, 0.512, 1.28, 3.2, 8, or 20 μM). Coumarin and bupropion were selected as specific substrates for CYP2A6 and CYP2B6, respectively. The substrates were used at a concentration approximately equal to their K_m_ values. (**e**) IC_50_ values and fold-shift in IC_50_ of (−)-POH and (−)-PAH after pre-incubation with or without NADPH. The activity is expressed as a percentage of remaining activity compared with that in the control containing no inhibitor. Data are presented as mean ± SD of values for three separate samples. Values given in parentheses represent 95% confidence intervals. * IC_50_ fold-shift = IC_50_ without NADPH/IC_50_ with NADPH.

To elucidate the mechanisms of inhibition of CYP2A6 and CYP2B6 by (−)-POH and (−)-PAH, their metabolism-dependent inhibition potentials were investigated (Figure 2). Metabolism-dependent inhibition was assessed by comparing the IC_50_ values observed in HLMs incubated with and without NADPH for 30 min. Metabolism-dependent inhibition is identified based on the IC_50_ shifts [21]. If the ratio of IC_50_ values observed in HLMs incubated without NADPH to those observed in HLMs incubated with NADPH exceeds 1.5, the test compound is classified as a metabolism-dependent inhibitor. This indicates that the metabolite(s) of the test compound exhibit more potent inhibitory activity against CYP enzymes than the parent compound. CYP2A6 activity was further decreased from 37.2% to 8.2% by 1 μM 8-methoxypsoralen, a positive metabolism-dependent inhibitor of CYP2A6, after pre-incubation with NADPH. CYP2B6 activity was also further decreased from 26.9% to 2.6% by 1 μM ticlopidine used as a positive metabolism-dependent inhibitor of CYP2B6 after pre-incubation in the presence of NADPH. The fold-shifts in IC_50_ of (−)-POH for CYP2A6 and CYP2B6 were 1.04 and 1.02, respectively (Figure 2a,b,e). The fold-shift in IC_50_ of (−)-PAH was 1.03 for CYP2A6 and 0.78 for CYP2B6 (Figure 2c,d,e). These results suggest that (−)-PAH may be metabolized to metabolite(s) with lower CYP2B6 inhibitory activity than (−)-PAH. Collectively, these data suggest that CYP inhibition by (−)-POH and (−)-PAH may not be metabolism-dependent.

### 3.3. Determination of Mode of Inhibition of CYP by (−)-POH and (−)-PAH

**Figure 3:**
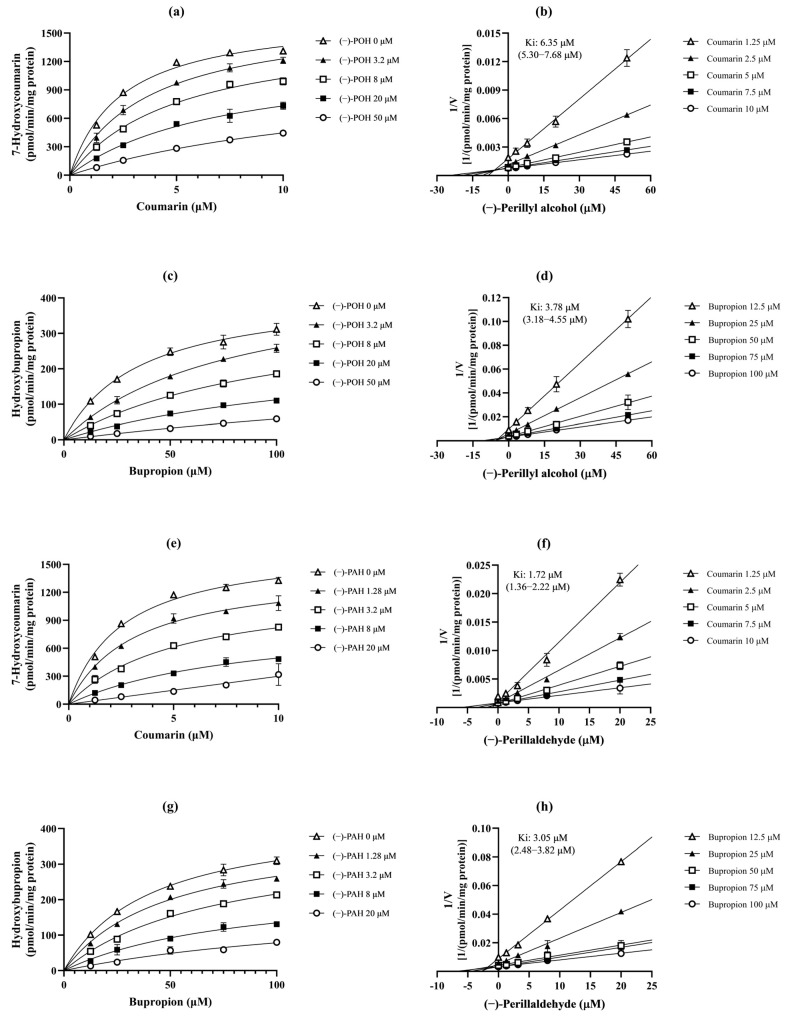
Michaelis–Menten plots (**a**,**c**,**e**,**g**) and Dixon plots (**b**,**d**,**f**,**h**) showing the inhibitory effects of (−)-POH and (−)-PAH on CYP2A6-mediated coumarin 7-hydroxylation and CYP2B6-mediated bupropion hydroxylation in pooled HLMs. Coumarin (1.25–10 μM) or bupropion (12.5–100 μM) were incubated with (−)-POH (0–50 μM) or (−)-PAH (0–20 μM) to determine the inhibitory constant (K_i_) and inhibition mode of (−)-POH and (−)-PAH from Dixon plots. Data are presented as mean ± SD of values for three separate samples. Values given in parentheses represent 95% confidence intervals.

To determine the inhibition type and K_i_ values for CYP2A6 and CYP2B6 inhibited by (−)-POH and (−)-PAH, various concentrations of substrate (coumarin for CYP2A6 or bupropion for CYP2B6) and inhibitor (−)-POH or (−)-PAH) were incubated with pooled HLMs. Substrate and inhibitor concentrations were chosen based on each substrate’s K_m_ value and the IC_50_ values of (−)-POH or (−)-PAH (Figure 3 and Table S4). An inhibitor can bind to the active site of an enzyme (competitive inhibition), to the enzyme–substrate complex (uncompetitive inhibition), or to both (mixed or noncompetitive inhibition). Among the estimated inhibition models, the model with the smallest AICc and Sy.x values was considered the best fit. AICc is an estimator of model prediction error, aiding in model selection, while Sy.x, the standard deviation of the residuals, indicates model fit quality. Dixon plots indicated that a mixed inhibition model was the best fit for the inhibition of CYP2A6 and CYP2B6 by (−)-POH and (−)-PAH. The K_i_ (95% CI) value of (−)-POH was 6.35 μM (5.30–7.68 μM) for CYP2A6 and 3.78 μM (3.18–4.55 μM) for CYP2B6 (Figure 3b,d). The K_i_ (95% CI) values of (−)-PAH against CYP2A6 and CYP2B6 were 1.72 μM (1.36–2.22 μM) and 3.05 μM (2.48–3.82 μM), respectively (Figure 3f,h).

### 3.4. Effect of POH on the Kinetic Parameters for MDZ Hydroxylation in Pooled HLMs

**Figure 4:**
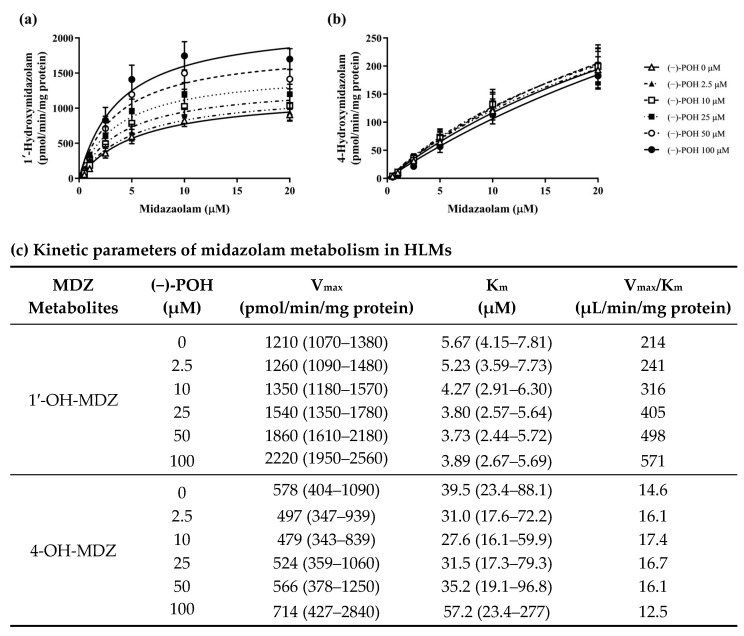
Effect of (−)-POH on the kinetic parameters obtained from the Michaelis–Menten model for the formation of 1′-OH-MDZ and 4-OH-MDZ in pooled HLMs. (**a**,**b**) MDZ was incubated at varying concentrations from 0.5 to 20 μM in the absence (△) or presence of 2.5 (▲), 10 (□), 25 (■), 50 (○), and 100 μM (●) (−)-POH. Data are expressed as mean ± SD of values for four separate samples. (**c**) Values given in parentheses represent 95% confidence intervals.

To elucidate the activation of CYP3A4 by (−)-POH, the kinetic studies of MDZ hydroxylation were performed with pooled HLMs (Figure 4). In the Michaelis–Menten model, the V_max_ values for MDZ 1′-hydroxylation were increased in a concentration-dependent manner with (−)-POH. Simultaneously, the K_m_ values for MDZ 1′-hydroxylation were decreased (Figure 4a,c). In contrast, increasing (−)-POH concentrations did not significantly affect the K_m_ and V_max_ values for MDZ 4-hydroxylation (Figure 4b,c). The V_max_/K_m_ value of MDZ 1′-hydroxylation, but not MDZ 4-hydroxylation, was increased 2.67-fold at 100 μM (−)-POH (571 μL/min/mg protein) compared with its value in the absence of (−)-POH (214 μL/min/mg protein). In the sigmoidal model, V_max_, Hill slope, and K_half_ (the concentration required to achieve half-maximal velocity) values for MDZ 1′-hydroxylation were also observed in a concentration-dependent manner with (−)-POH (Figure S3).

### 3.5. Effect of POH on MDZ 1′-Hydroxylation in the Absence or Presence of Cytochrome b_5_ in rCYP3A4

**Figure 5:**
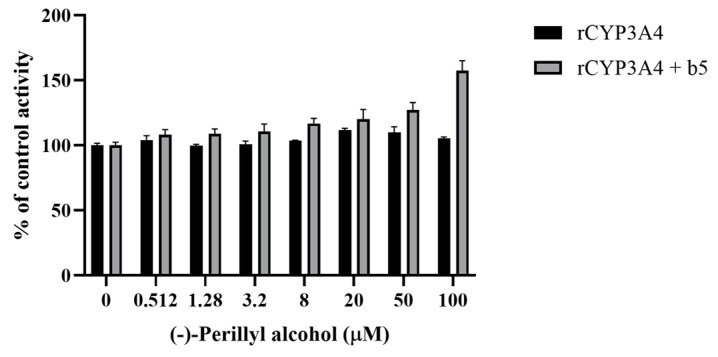
Effect of (−)-POH on MDZ 1′-hydroxylation in rCYP3A4 in the absence or presence of cytochrome *b*_5_. The activity is expressed as a percentage of remaining activity compared with that for the control containing no (−)-POH. Data are presented as mean ± SD of values for three separate samples.

We further investigated the activation effect of (−)-POH on MDZ hydroxylation using rCYP3A4, both with and without cytochrome *b*_5_. rCYP3A4 enzyme is prepared from baculovirus-transfected insect cells, which exhibit higher activity compared to HLMs. Using rCYP3A4 helps to exclude metabolic effects from other CYP isoforms and non-CYP enzymes in pooled HLMs. CYP enzyme activity and cooperativity are modulated by cytochrome *b*_5_ [17,18,19,20]. The activation of CYP3A4-mediated MDZ 1′-hydroxylation by (−)-POH was observed in rCYP3A4 in the presence of cytochrome *b*_5_ (Figure 5). The CYP3A4 activity for 1′-OH-MDZ formation was increased 1.57-fold at 100 μM (−)-POH compared with that for the control without (−)-POH.

### 3.6. Effect of POH on MDZ Hydroxylation in hHOs

**Figure 6:**
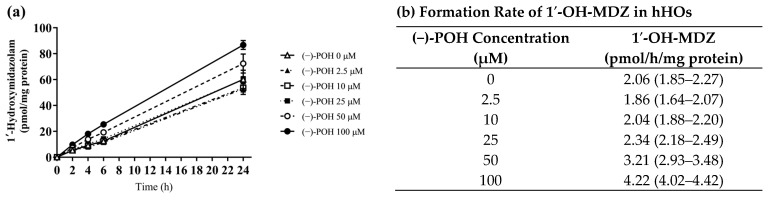
Effect of (−)-POH on MDZ 1′-hydroxylation in hHOs. (**a**) MDZ (5 μM) was incubated in the absence (△) or presence of 2.5 (▲), 10 (□), 25 (■), 50 (○), and 100 μM (●) (−)-POH for 0, 2, 4, 6, or 24 h. Data are presented as mean ± SD of values for four separate samples. (**b**) The rate of 1′-OH-MDZ formation was calculated up to 6 h of incubation only in the linear section. Values given in parentheses represent a 95% confidence interval.

To confirm the enhancement of CYP3A4-mediated MDZ 1′-hydroxylation, but not 4-hydroxylation, by POH, hHOs were treated with 5 μM MDZ in the presence of various concentrations of (−)-POH (Figure 6). The amount of 1′-OH-MDZ produced in hHOs was plotted against the incubation time (Figure 6a). Linearity was observed up to 6 h of incubation. Therefore, the 1′-OH-MDZ formation rate was calculated up to 6 h of incubation. The rate of 1′-OH-MDZ formation was increased 2.05-fold at 100 μM (−)-POH (4.22 pmol/h/mg protein) compared with the rate in the absence of (−)-POH (2.06 pmol/h/mg protein) (Figure 6b). However, the rate of 4-OH-MDZ formation was not significantly different between the control and POH-treated hHOs. The formation rate of 4-OH-MDZ was 0.220 ± 0.061 pmol/h/mg protein in the absence of (−)-POH. At the highest concentration of 100 μM (−)-POH treatment, the for-mation rate of 4-OH-MDZ was 0.159 ± 0.016 pmol/h/mg protein.

### 3.7. Effect of POH on Metabolism of Other CYP3A4 Substrates in Pooled HLMs

**Figure 7:**
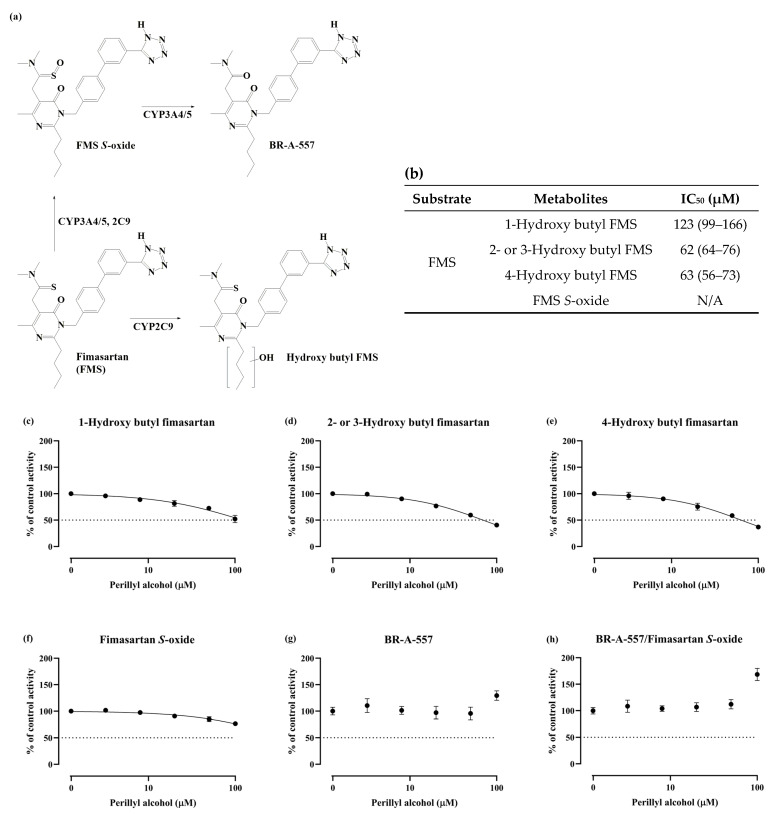
Effects of (−)-POH on fimasartan (FMS) metabolism in pooled HLMs. (**a**) CYP-mediated metabolic scheme of FMS adapted from Choi et al. [27]. (**b**) IC_50_ values of (−)-POH against CYP-mediated FMS metabolism. (**c**–**h**) FMS (30 μM) was incubated for 10 min in the absence or presence of (−)-POH (3.2, 8, 20, 50, or 100 μM) in HLMs. The activity is expressed as a percentage of remaining activity compared with that in the control containing no inhibitor. Data are presented as mean ± SD of values for three separate samples. N/A, not applicable.

To investigate the effect of (−)-POH on other CYP3A4 substrates, the metabolism of atorvastatin, buspirone, donepezil, FMS, nifedipine, and tadalafil as CYP3A4 substrates was determined in pooled HLMs. (−)-POH did not significantly affect the CYP3A4-mediated metabolism of atorvastatin, buspirone, donepezil, nifedipine, and tadalafil (Figure S4). (−)-POH inhibited the CYP2C9-mediated formation of 1-hydroxy butyl FMS, 2- or 3-hydroxy butyl FMS, and 4-hydroxy butyl FMS from FMS (Figure 7c–e). BR-A-557, which is produced from FMS by CYP3A4, increased 1.29-fold at 100 μM (−)-POH relative to the control (Figure 7g). The increased formation of BR-A-557 by (−)-POH was more pronounced when expressed as the ratio of BR-A-557 to FMS *S*-oxide, a metabolic intermediate of BR-A-557 (Figure 7h). The formation of FMS *S*-oxide by CYP3A4 and CYP2C9 was reduced by (−)-POH (Figure 7f). Inhibition of the formation of hydroxy FMS at *n*-butyl group and FMS *S*-oxide by (−)-POH was consistent with the results showing CYP2C9 inhibition in this study (Figure 1e).

## 4. Discussion

In the present study, we aim to investigate the CYP-mediated drug–drug interaction potential of POH and its precursor and metabolites. First, we performed a direct CYP inhibition assay using pooled HLMs. The results showed that the IC_50_ values of (+)-PAH against CYP2A6, CYP2B6, and CYP2C19 were approximately three- to five-fold higher than those of (−)-form of PAH (Table 1). These results indicate that the (−)-form of PAH could be a more potent inhibitor than the (+)-form. Based on these findings, CYP inhibition studies were performed on the precursors and metabolites of PAH, focusing on the (−) form. (−)-POH, a precursor of PAH, inhibited CYP2A6 and CYP2B6 (Table 1). (−)-POH also inhibited the activities of CYP2C9, CYP2C19, and CYP2E1, with IC_50_ values higher than 50 μM (Table 1). These results indicate that POH and PAH can significantly inhibit CYP2A6 and CYP2B6 activities. In comparison, both the (+) and (−) forms of LMN and (−)-PA did not significantly inhibit any of the CYP isoforms (Table 1). Interestingly, in the direct inhibition assay, we discovered that (−)-POH not only inhibited CYP2A6 and CYP2B6 but also stimulated the CYP3A4 activity. Especially, the activation of CYP3A4 by (−)-POH was only observed in HLMs incubated with MDZ, but not in testosterone as a substrate.

The metabolism- or time-dependent inhibition can be evaluated by comparing the IC_50_ values for pre-incubation in the absence and presence of NADPH [29]. A metabolism-dependent inhibitor exhibits a lower IC_50_ value observed in pre-incubation with NADPH than without NADPH, suggesting that a metabolite(s) of the test compound has a more potent inhibitory activity against CYP enzymes than the parent compound. Compounds with IC_50_-fold shifts greater than 1.5 can be considered significant metabolism-dependent inhibitors [30]. Our results indicated IC_50_-fold shift values of less than 1.5 for both CYP2A6-mediated coumarin 7-hydroxylation and CYP2B6-mediated bupropion hydroxylation by (−)-POH and (−)-PAH. These results indicate that CYP inhibition by (−)-POH and (−)-PAH may not be metabolism-dependent (Figure 2). Generally, it was anticipated that the IC_50_ value obtained after pre-incubation for 30 min without NADPH and that obtained in direct inhibition assay would be similar. However, the IC_50_ values of (−)-POH and (−)-PAH against CYP2A6 and CYP2B6 obtained after pre-incubation with NADPH for 30 min were approximately double those obtained in direct inhibition assay performed without pre-incubation with NADPH (Table 1 and Figure 2). NADPH-dependent or NADPH-independent microsomal metabolism of a test compound can decrease its concentration, leading to elevation of IC_50_ values. On the other hand, POH and its metabolism-related compounds are known to have volatile properties as monoterpenes [7,31]. Thus, it is presumed that the difference between the IC_50_ values between direct inhibition and metabolism-dependent inhibition assays may be due to the volatilization of the semi-volatile organic compounds (−)-POH and (−)-PAH.

Reversible inhibition can be categorized as competitive, noncompetitive, uncompetitive, and mixed inhibition. The K_i_ values of (−)-POH against CYP2A6 and CYP2B6 were 6.35 and 3.78 μM, respectively, and the inhibition type was the best fit for the mixed inhibition model (Figure 3 and Table S4). The inhibition type of (−)-PAH for CYP2A6 and CYP2B6 was the best fit for the mixed inhibition model, with K_i_ values of 1.72 and 3.05 μM, respectively (Figure 3 and Table S4). These results indicate that (−)-PAH has a more potent inhibitory affinity for CYP2A6 than (−)-POH.

Heterotropic activation of CYP3A4 can be demonstrated by an increase in V_max_, a decline in K_m_, or both [32]. In the kinetic analysis using pooled HLMs, we observed an appreciable increase in V_max_ and a decrease in K_m_ values for 1′-OH-MDZ in a POH concentration-dependent manner (Figure 4). However, no considerable alteration in the kinetic parameter values was observed for the 4-hydroxylation of MDZ in response to POH concentrations. These results indicate that the metabolism of MDZ 1′-hydroxylation, but not 4-hydroxylation, may be stimulated by POH through heterotropic activation of CYP3A4 (Figure 4). Huang et al. [33] first demonstrated the activation of benzopyrene hydroxylation by flavone and 7,8-benzoflavone in reconstituted rabbit CYP enzymes. Additionally, Ueng et al. [34] reported heterotropic cooperativity by 7,8-benzoflavone in purified recombinant CYP3A4. Furthermore, evidence from X-ray structural analysis suggests that the active site of CYP3A4 can accommodate more than one substrate, such as ketoconazole and ritonavir [35,36]. Although the mechanism of CYP cooperativity remains poorly understood, several models have been suggested to explain it. The “space-filling model” was an early hypothesis explaining heterotropic cooperativity [37]. It is premised on the assumption that broad substrate specificity results in large substrate binding pockets that can bind more than one ligand simultaneously. However, in the absence of structural evidence, demonstrating that two ligand molecules exist adjacent to each other is difficult [38]. “Nested allosterism”, which combines the “space-filling” model with the classical concept of allosterism, postulating the existence of two distinct binding sites, was subsequently proposed by Atkins and colleagues as a hypothesis to explain CYP cooperativity [39]. In addition, Davydov and colleagues suggested a model combining multiple substrate binding, ligand-induced conformational transitions, and ligand-modulated persistence heterogeneity to explain the heterotropic cooperativity of CYP3A4 [40].

Our results show that cytochrome *b*_5_ is required to activate MDZ 1′-hydroxylation by POH (Figure 5). No significant enhancement by POH was observed in rCYP3A4 in the absence of cytochrome *b*_5_ (Figure 5). Several studies have reported cytochrome *b*_5_-depenent heterotropic activation. The activation of CYP3A4-mediated MDZ metabolism by efavirenz was indicated to depend on the presence of cytochrome *b*_5_ [18]. The presence of cytochrome *b*_5_ was also reported to influence the enhancement in CYP3A4-mediated MDZ activity by VU0448187, a positive allosteric modulator of metabotropic glutamate receptor 5 [19]. Similarly, Zhuang et al. [20] demonstrated that the activation of CYP3A5-mediated MDZ metabolism by icotinib requires the presence of cytochrome *b*_5_. Although the precise mechanism by which cytochrome *b*_5_ contributes to the activation of MDZ 1′-hydroxylation by POH remains unclear, two potential explanations have been proposed. One hypothesis suggests that cytochrome *b*_5_ induces a conformational change in CYP enzymes [41,42], whereas another posits that cytochrome *b*_5_ enhances the coupling between CYP and reductase [43,44].

To ascertain whether the same phenomenon is evident in living cells, an additional experiment was conducted using human pluripotent stem cell-derived hHOs. The results indicated that the formation of 1′-OH-MDZ but not 4-OH-MDZ in hHOs increased in a POH concentration-dependent manner (Figure 6). The results from pooled HLMs and hHOs suggest that the heterotropic activation of CYP3A4 by POH is regioselective. Similar regioselective activation of CYP3A4 was observed in HLM and rCYP3A4 treated with tangeretin [45], although the underlying mechanism remains unidentified.

Finally, we investigated the effect of POH on the metabolism of CYP3A4 substrates other than MDZ. Atorvastatin, buspirone, donepezil, FMS, nifedipine, and tadalafil are known to be metabolized by CYP3A4 [22,23,25,27,46,47], although the contribution of CYP3A4 to their overall clearance is variable. Among the drugs tested, the formation of BR-A-557 from FMS was only increased by POH (Figure 7). Choi et al. [27] demonstrated that both CYP2C9 and CYP3A4 play significant roles in the formation of FMS *S*-oxide from FMS; however, the formation of BR-A-557 from FMS *S*-oxide is mediated by CYP3A4 but not by CYP2C9. The hydroxylation of the *n*-butyl group of FMS to produce 1-, 2-, 3-, and 4-hydroxy butyl FMS is primarily mediated by CYP2C9 [27]. Our results showed that the formation of BR-A-557 was increased by POH (Figure 7), although the formation of FMS *S*-oxide, a metabolic intermediate of BR-A-557, was decreased by POH (Figure 7). The decreased formation of FMS *S*-oxide and hydroxy butyl FMS metabolites is consistent with the results from the direct CYP inhibition, which showed that the CYP2C9 activity was decreased to 64.2% of the control level by 50 μM POH.

In a phase I clinical trial of POH, 17 patients took POH orally three times daily for 14 days, with starting doses of 1600 mg/m^2^/dose, escalating to 2100 and 2800 mg/m^2^/dose [48]. In the previous study, dihydroperillic acid and PA, but not POH, were measured in plasma and urine. The peak plasma concentrations of PA, the major metabolite, and dihydroperillic acid ranged from 433.2 μM to 774.1 μM and from 22.6 μM to 42.4 μM, respectively. These results suggest that a considerable amount of POH is absorbed by the gastrointestinal tract and rapidly metabolized to PA. Western blot analysis using selective CYP antibodies revealed that CYP3A (CYP3A4 and CYP3A5) constitutes approximately 80% of the total immunoquantified CYP content, emphasizing its significant role in intestinal drug metabolism [49]. Previous studies have demonstrated that normal consumption of grapefruit juice inhibits intestinal CYP3A without affecting hepatic CYP3A, thereby increasing the bioavailability of CYP3A substrates [50]. This study underscores the importance of further evaluation of the metabolic interactions between POH and drugs metabolized by gastrointestinal CYP enzymes in clinical situations to optimize therapeutic outcomes.

## 5. Conclusions

In the present study, we evaluate the effect of (−)-POH and its precursor and metabolites on CYP activities to understand possible drug interactions. CYP2A6 and CYP2B6 were inhibited by (−)-POH and (−)-PAH, and the type of inhibition showed a best fit to mixed inhibition. Moreover, the metabolism of MDZ 1′-hydroxylation, but not 4-hydroxylation, may be stimulated by (−)-POH through heterotropic activation of CYP3A4. Although it is not clearly understood how cytochrome *b*_5_ contributes to heterotropic cooperativity, we found that the activation occurs in a cytochrome *b*_5_-dependent manner. Moreover, the heterotropic activation of CYP3A4 by (–)-POH was also observed in human pluripotent stem cell-derived hHOs. Activation of CYP3A4-mediated metabolism was observed in HLMs incubated with FMS but not atorvastatin, buspirone, donepezil, nifedipine, or tadalafil. These results indicate that the activation of CYP3A4 by (−)-POH may be substrate-dependent. Based on the fact that CYP3A4 metabolizes more than 50% of clinically used drugs, further studies are warranted to assess the clinical drug interaction of (−)-POH with other CYP3A4 substrates.

## Figures and Tables

**Table 1:** IC_50_ values of (+)-LMN, (−)-LMN, (−)-POH, (+)-PAH, (−)-PAH, and (−)-PA against CYP isoforms in pooled HLMs.

CYP Isoforms	Substrate	IC_50_ (μM)					
		(+)-LMN	(−)-LMN	(+)-PAH	(−)-PAH	(−)-POH	(−)-PA
CYP1A2	Phenacetin	N/A	N/A	N/A	N/A	N/A	N/A
CYP2A6	Coumarin	N/A	N/A	10.6 (9.07–12.4)	3.64 (3.40–3.90)	15.7 (12.8–19.3)	N/A
CYP2B6	Bupropion	N/A	N/A	29.0 (23.6–36.6)	6.15 (5.53–6.85)	6.99 (5.82–8.41)	N/A
CYP2C8	Amodiaquine	N/A	N/A	N/A	N/A	N/A	N/A
CYP2C9	Tolbutamide	N/A	N/A	N/A	N/A	N/A	N/A
CYP2C19	(S)-Mephenytoin	N/A	N/A	50.5 (33.0–100)	18.0 (13.2–25.0)	N/A	N/A
CYP2D6	Dextromethorphan	N/A	N/A	N/A	N/A	N/A	N/A
CYP2E1	Chlorzoxazone	N/A	N/A	N/A	N/A	N/A	N/A
CYP3A4	Midazolam	N/A	N/A	N/A	N/A	Stimulation *	N/A
	Testosterone	N/A	N/A	N/A	N/A	N/A	N/A

Each test compound was incubated at 0, 0.512, 1.28, 3.2, 8, 20, or 50 μM in pooled HLMs. Values given in parentheses represent 95% confidence intervals. N/A, not applicable. * MDZ 1′-hydroxylation was increased by (−)-POH.

## Data Availability

The original contributions presented in this study are included in the article/Supplementary Material. Further inquiries can be directed to the corresponding authors.

## Supplementary Materials

The following supporting information can be downloaded at https://www.mdpi.com/article/10.3390/pharmaceutics16121581/s1: Table S1: Lower limit of quantitation (LLOQ) of CYP metabolites; Table S2: Analytic parameters for the mass spectrometric detection of analytes using API 4000 system; Table S3: IC_50_ values of k nown CYP isoform selective inhibitors; Table S4: CYP inhibition type and inhibition constant (K_i_) values of (−)-POH and (−)-PAH; Figure S1: Chemical structure of (+)-LMN (**a**), (−)-LMN (**b**), (+)-PAH (**c**), (−)-PAH (**d**), (−)-POH (**e**), and (−)-PA (**f**); Figure S2: Representative LC-MS/MS chromatograms of CYP-mediated metabolite standards and internal standards; Figure S3: Effect of (−)-POH on the kinetic parameters obtained from the sigmoidal model for the formation of 1′-OH-MDZ and 4-OH-MDZ in pooled HLMs; Figure S4: Effect of (−)-POH on the metabolism of CYP3A4 substrates in pooled HLMs.
