# Supplementary material for: Heterotropic Activation of Cytochrome P450 3A4 by Perillyl Alcohol
Source: Pharmaceutics. 2024 Dec 11;16(12):1581. doi: 10.3390/pharmaceutics16121581 (PMC11676982; doi:10.3390/pharmaceutics16121581)
Supplement: Supplementary file 1 [file pharmaceutics-16-01581-s001.zip › pharmaceutics-3324052-supplementary.pdf]

pharmaceutics

Supplementary Materials:

Heterotropic Activation of Cytochrome P450 3A4 by Perillyl Alcohol

Ji Hyeon Ryu, Jieun Yu, Jang Su Jeon, Seongyea Jo, Soo Min Lee, Hyemin Kim, Han-Jin Park, Soo Jin Oh, and Sang Kyum Kim

1. Supplementary Tables

Table S1. Lower limit of quantification (LLOQ) of CYP metabolites

**Analytes**	**LLOQ (nM)**
Acetaminophen	15.6
7-Hydroxycoumarin	3.9
Hydroxybupropion	14.2
N-Desethylamodiaquine	3.9
4-Hydroxytolbutamide	3.7
4-Hydroxymephenytoin	3.9
Dextrorphan	15.6
6-Hydroxychlorzoxazone	167
1´-Hydroxymidazolam	1.8
4-Hydroxymidazolam	1.6
6β-Hydroxytestosterone	9.0

Table S2. Analytic parameters for the mass spectrometric detection of analytes using API 4000 system.

Analytes	Transition (m/z)	Retention Time (min)	Declustering Potential (mV)	Collision Energy (mV)	Mode
Acetaminophen	152.1→110.1	2.40	56	23	ESI+
7-Hydroxycoumarin	163.0→107.0	2.57	53	35	ESI+
Hydroxybupropion	256.0→238.0	2.40	41	15	ESI+
N-Desethylamodiaquine	328.1→283.2	2.37	61	23	ESI+
4-Hydroxytolbutamide	287.1→170.9	2.61	61	23	ESI+
4-Hydroxymephenytoin	235.1→150.0	2.51	68	24	ESI+
Dextrorphan	258.2→157.0	2.40	80	47	ESI+
6-Hydroxychlorzoxazone	183.8→120.0	2.51	-50	-26	ESI-
1´-Hydroxymidazolam	342.1→324.2	2.47	95	35	ESI+
4-Hydroxymidazolam	342.3→234.0	2.95	50	30	ESI+
6β-Hydroxytestosterone	305.3→269.1	2.68	61	19	ESI+
1-Hydroxy butyl fimasartan	518.2→207.2	3.48	60	40	ESI+
2- or 3-Hydroxy butyl fimasartan	518.2→207.2	2.97	60	40	ESI+
4-Hydroxy butyl fimasartan	518.2→207.2	2.90	60	40	ESI+
Fimasartan *S*-oxide	518.2→207.2	3.10	60	40	ESI+
BR-A-557	486.2→207.2	3.28	41	35	ESI+
ortho-Hydroxyatorvastatin	575.0→466.0	4.56	80	20	ESI+
para-Hydroxyatorvastatin	575.0→440.0	3.34	50	30	ESI+
6´-Hydroxybuspirone	402.0→122	2.74	80	50	ESI+
Desbenzyl donepezil	290.0→273.3	7.60	81	25	ESI+
Donepezil *N*-oxide	396.2→91.2	10.62	81	59	ESI+
Oxidized nifedipine	345.0→284.0	3.23	50	35	ESI+
Desmethylene tadalafil	378.1→256.2	11.06	101	23	ESI+
Carbamazepine	237.2→256.2	2.84	56	29	ESI+
4-Methylumbelliferone	174.9→256.2	2.66	-70	-30	ESI-

ESI+: positive electrospray ionization; ESI-: negative electrospray ionization

Table S3. IC_{50} values of known CYP isoform selective inhibitors

CYP Isoforms	Substrate	Inhibitor	IC_{50} (μM)
CYP1A2	Phenacetin	Fluvoxamine	0.31
CYP2A6	Coumarin	Tranylcypromine	0.50
CYP2B6	Bupropion	Paroxetine	1.20
CYP2C8	Amodiaquine	Quercetin	5.20
CYP2C9	Tolbutamide	Sulfaphenazole	0.51
CYP2C19	(S)-Mephenytoin	Omeprazole	5.40
CYP2D6	Dextromethorphan	Quinidine	0.08
CYP2E1	Chlorzoxazone	4-Methylpyrazole	1.00
CYP3A4	Midazolam	Ketoconazole	0.12
	Testosterone	Ketoconazole	0.09

Table S4. CYP inhibition type and inhibition constant (K_i) values of (−)-POH and (−)-PAH

Compounds	CYP Isoforms	Type of Inhibition	K_i [a] (μM)	R^2	AICc[b]	Sy.x[c]	Selection
(-)-POH	CYP2A6	Competitive	4.91 (4.43–5.43)	0.9897	553.8	38.7	
		Noncompetitive	18.5 (17.0–20.3)	0.9793	606.2	54.9	
		Uncompetitive	10.9 (9.16–12.9)	0.9454	678.9	89.0	
		Mixed	6.35 (5.30–7.68)	0.9912	544.5	36.1	✓
	CYP2B6	Competitive	3.36 (3.06–3.69)	0.9921	315.8	7.92	
		Noncompetitive	9.70 (8.96–10.5)	0.9836	370.9	11.4	
		Uncompetitive	4.54 (3.62–5.56)	0.9500	454.6	20.0	
		Mixed	3.78 (3.18–4.55)	0.9924	315.6	7.84	✓
(-)-PAH	CYP2A6	Competitive	1.05 (0.91–1.21)	0.9815	601.2	53.1	
		Noncompetitive	4.04 (3.72–4.40)	0.9799	607.2	55.2	
		Uncompetitive	2.40 (2.04–2.82)	0.9504	675.1	86.8	
		Mixed	1.72 (1.36–2.22)	0.9868	577.9	45.0	✓
	CYP2B6	Competitive	2.00 (1.79–2.23)	0.9875	347.1	9.75	
		Noncompetitive	5.73 (5.34–6.15)	0.9870	349.9	9.93	
		Uncompetitive	2.77 (2.31–3.28)	0.9600	434.3	17.4	
		Mixed	3.05 (2.48–3.82)	0.9907	327.4	8.48	✓

Values given in parentheses represent 95% confidence intervals.
[a]Ki for inhibition constant
[b]AICc, Akaike's information criterion with correction for small sample size
[c]Sy.x, Standard deviation of the residuals

2. Supplementary Figures

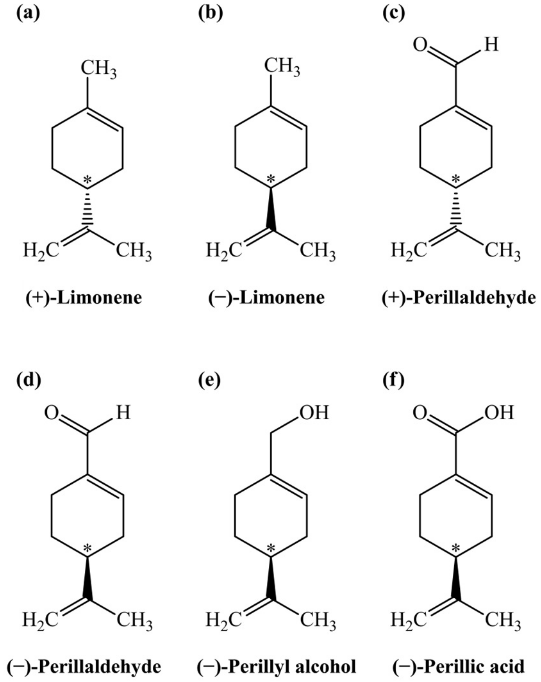

Figure S1. Chemical structure of (+)-LMN (**a**), (−)-LMN (**b**), (+)-PAH (**c**), (−)-PAH (**d**), (−)-POH (**e**), and (−)-PA (**f**). *, Chiral center

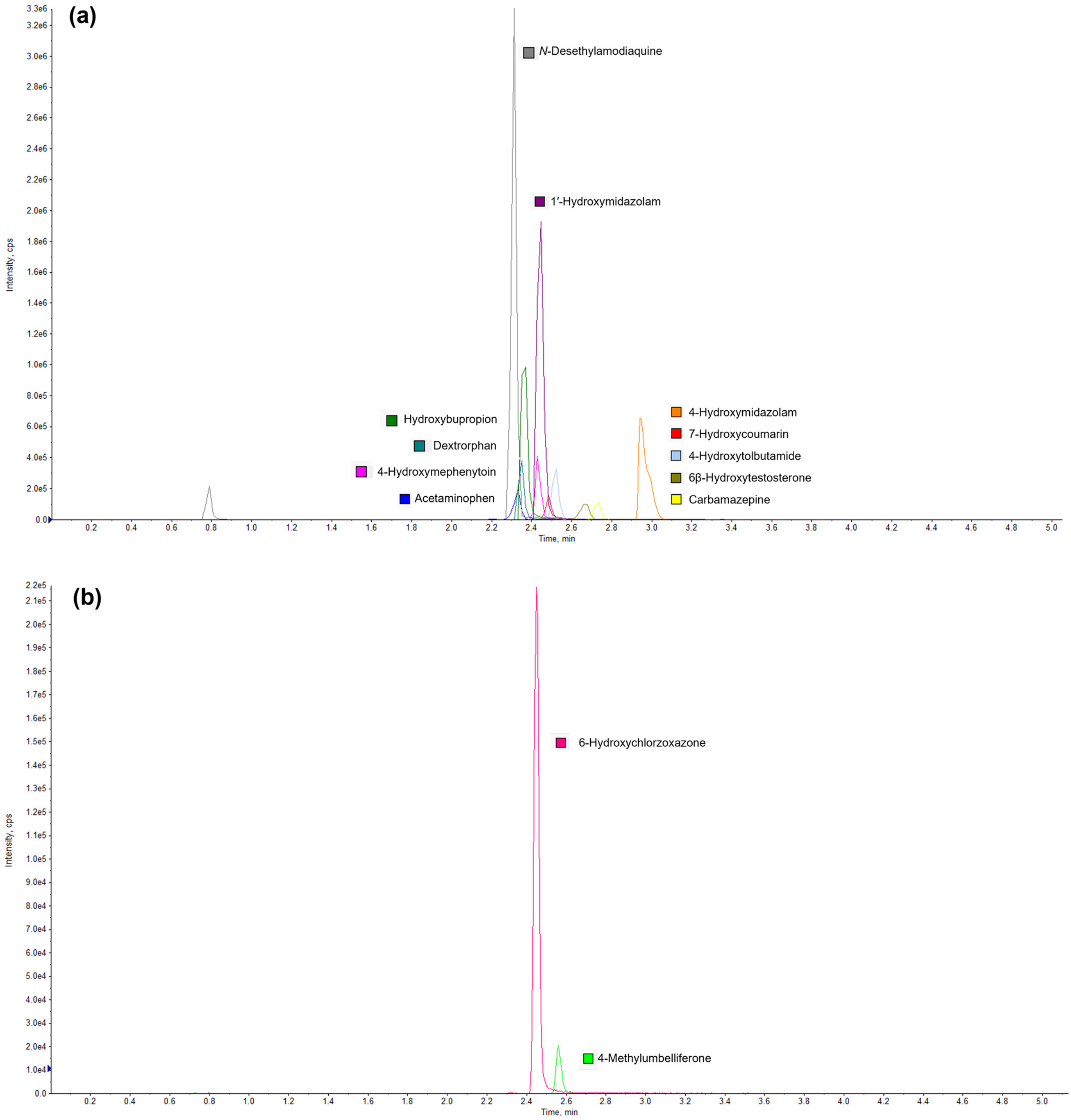

Figure S2. Representative LC-MS/MS chromatograms of CYP-mediated metabolite standards and internal standards. (**a**) Chromatogram obtained in ESI+ mode. (**b**) Chromatogram obtained in ESI- mode.

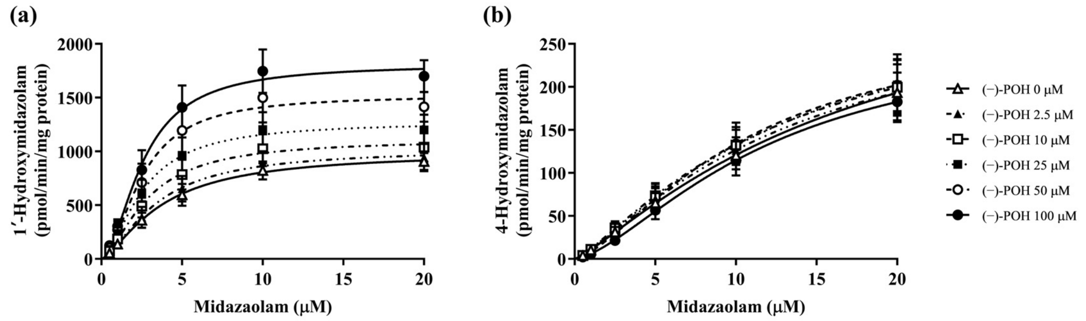

(c) Kinetic parameters of midazolam metabolism in HLMs

MDZ metabolites	(−)-POH (μM)	V_{max} (pmol/min/mg protein)	h	K_{half}[b] (μM)
1′-OH-MDZ	0	994 (891–1166)	1.45 (1.10–1.89)	3.68 (2.92–5.20)
	2.5	1022 (906–1223)	1.55 (1.10–2.18)	3.36 (2.60–4.94)
	10	1111 (996–1290)	1.61 (1.15–2.28)	2.82 (2.21–3.91)
	25	1275 (1148–1463)	1.66 (1.19–2.36)	2.56 (2.01–3.47)
	50	1523 (1375–1727)	1.83 (1.28–2.71)	2.51 (1.99–3.28)
	100	1808 (1659–2006)	1.83 (1.35–2.52)	2.58 (2.12–3.20)
4-OH-MDZ	0	352 (238–2623)	1.13 (0.845–1.67)	17.0 (9.04–375)
	2.5	307 (215–1976)	1.28 (0.822–1.86)	13.2 (7.35–276)
	10	307 (219–1427)	1.28 (0.825–1.86)	12.4 (7.03–168)
	25	326 (223–7181)	1.27 (0.791–1.88)	13.6 (7.28–1579)
	50	282 (214–688)	1.48 (0.971–2.13)	10.7 (6.98–46.9)
	100	266 (204–581)	1.53 (1.07–2.10)	12.0 (8.12–40.0)

Figure S3. Effect of (−)-POH on the kinetic parameters obtained from the sigmoidal model for the formation of 1′-OH-MDZ and 4-OH-MDZ in pooled HLMs. (**a**,**b**) MDZ was incubated at varying concentrations from 0.5 to 20 μM in the absence (△) or presence of 2.5 (▲), 10 (□), 25 (■), 50 (○), and 100 μM (●) (−)-POH. Data are expressed as mean ± SD of values for four separate samples. (**c**) Values given in parentheses represent 95% confidence intervals. [a]h, Hill slope; [b]K_{half} for the ligand concentration required to achieve half-maximal velocity

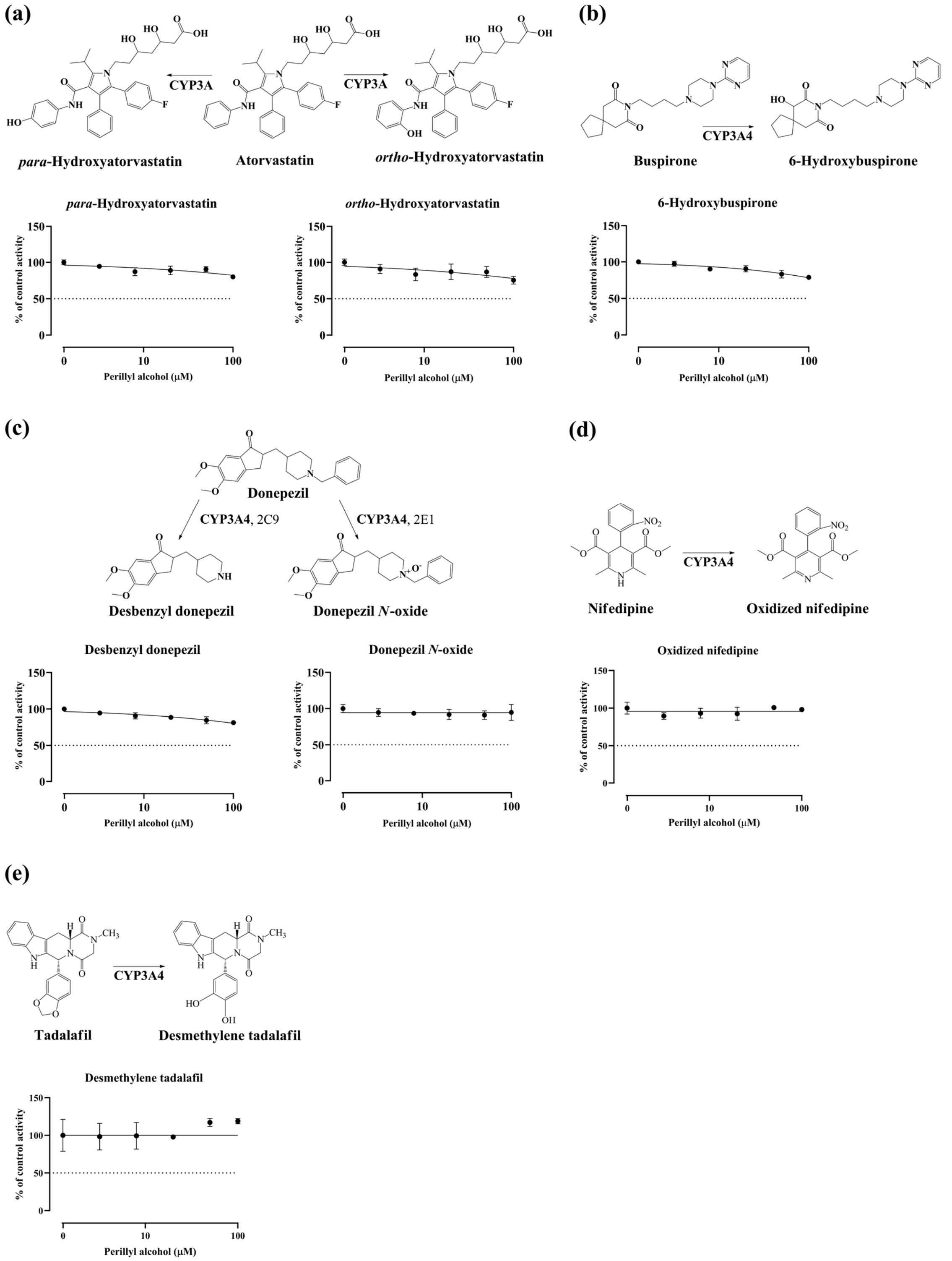

Figure S4. Effect of (−)-POH on the metabolism of CYP3A4 substrates in pooled HLMs. Atorvastatin (**a**, 30 μM), buspirone (**b**, 8 μM), donepezil (**c**, 35 μM), nifedipine (**d**, 25 μM), or tadalafil (**e**, 5 μM) was incubated in the absence or presence of (-)-POH (3.2, 8, 20, 50, or 100 μM). The substrates were used at a concentration approximately equal to their K_m values. The activity is expressed as the percentage of remaining activity compared with that in the control containing no inhibitor. Data are presented as mean ± SD of values for three separate samples.
